# *Angiopteris cochinchinensis* de Vriese Ameliorates LPS-Induced Acute Lung Injury via Src Inhibition

**DOI:** 10.3390/plants11101306

**Published:** 2022-05-13

**Authors:** Won Young Jang, Hwa Pyoung Lee, Seung A Kim, Lei Huang, Ji Hye Yoon, Chae Yun Shin, Ankita Mitra, Han Gyung Kim, Jae Youl Cho

**Affiliations:** 1 Department of Integrative Biotechnology, Sungkyunkwan University, Suwon 16419, Korea; wybest0327@naver.com (W.Y.J.); leehwapyoung57@gmail.com (H.P.L.); seung-a26@naver.com (S.A.K.); 2 Department of Biocosmetics, Sungkyunkwan University, Suwon 16419, Korea; ruidan0909@naver.com (L.H.); kws05251@naver.com (J.H.Y.); shina8059@naver.com (C.Y.S.); 3 Department of Integrative Biotechnology and Biomedical Institute for Convergence at SKKU (BICS), Sungkyunkwan University, Suwon 16419, Korea; ankitamitra1710@gmail.com

**Keywords:** *Angiopteris cochinchinensis* de Vriese, LPS, acute lung injury, NF-κB, Src, anti-inflammation

## Abstract

Growing demand for treatment options against acute lung injury (ALI) emphasizes studies on plant extracts harboring anti-inflammatory effects. According to GC-MS analysis, *Angiopteris cochinchinensis* de Vriese consists of various flavonoids with anti-inflammatory activities. Thus, in this study, the anti-inflammatory effects of an extract of *Angiopteris cochinchinensis* de Vriese (Ac-EE) were assessed using RAW264.6 murine macrophages and a lipopolysaccharide (LPS)-induced ALI model. Ac-EE reduced the nitric oxide production in murine macrophages increased by LPS induction. Moreover, protective effects of Ac-EE on lung tissue were demonstrated by shrinkage of edema and lung injury. Reduced neutrophil infiltration and formation of hyaline membranes were also detected in lung tissues after H&E staining. Semiquantitative RT-PCR, quantitative real-time PCR, and ELISA showed that Ac-EE inhibits the production of proinflammatory mediators, including *i*NOS and COX-2, and cytokines, such as TNF-α, IL-1β, and IL-6. An Ac-EE-mediated anti-inflammatory response was derived from inhibiting the NF-κB signaling pathway, which was evaluated by luciferase reporter assay and Western blotting analysis. A cellular thermal shift assay revealed that the prime target of Ac-EE in alleviating inflammation was Src. With its direct binding with Src, *Angiopteris cochinchinensis* de Vriese significantly mitigates lung injury, showing possibilities of its potential as an effective botanical drug.

## 1. Introduction

Acute lung injury (ALI) is a life-threatening disorder that leads to cardiogenic pulmonary edema and arterial hypoxemia [1]. Among various factors, the top-ranked etiology of ALI derives from bacterial or viral infections causing an inflammatory response in the lung [2,3,4]. Lung inflammation progresses through neutrophil infiltration and formation of hyaline membranes and eventually damages the tissues of the breathing system [5]. Since severe morbidity and mortality of ALI have been reported [6], clinical trials of various therapies, such as corticosteroids or neutrophil elastase inhibitor, have been conducted, but most of them failed to achieve a reduction in mortality rate [7]. Thus, there is a huge demand for novel therapeutic agents against ALI with enhanced treatment effects.

The inflammatory response against specific pathogens is initiated as the immune cells recognize pathogen-associated molecular patterns [8]. The response can be mediated by pattern recognition receptors (PRRs) with ligand–receptor interactions [9]. Most widely reported cases include interaction of lipopolysaccharide (LPS), Pam3csk4, and Poly (I:C) with toll-like receptor 4 (TLR4), TLR1/2, and TLR3, respectively [10,11,12]. LPS is a cell wall component of Gram-negative bacteria, such as *Pseudomonas aeruginosa*, that contributes to bacterial pneumonia, causing severe lung injury [13,14]. Poly (I:C), which has an analogous structure to viral dsDNA, and Pam3csk4, a lipopeptide found in the cell walls of both Gram-positive and Gram-negative bacteria, can also be the causes of inflammation-derived diseases [15,16].

Protein kinases mediate cellular responses via phosphorylation of threonine, tyrosine, or serine residues utilizing the phosphate group of ATP. Among them, proto-oncogene tyrosine protein kinase Src (c-Src or Src) is known to participate in various biological responses, such as cell metabolism, cell adhesion, and cell survival [17,18,19]. It also acts as a crucial innate immunity mediator, as the stimulus from TLRs activated by PRR engagement is transmitted to downstream molecules, such as TIR1-domain-containing adapter-inducing interferon-β (TRIF), delivering signals to Src [20]. This leads to phosphorylation of Src and downstream proteins, such as phosphoinositide 3-kinase (PI3K), protein kinase B (AKT), and NF-κB-inhibitor alpha (IκBα) [21,22]. Among them, IκBα phosphorylation results in its degradation, releasing nuclear factor kappa B (NF-κB) [23].

NF-κB activation accompanied by translocation to the nucleus leads to expression of proinflammatory cytokines, including tumor necrosis factor alpha (TNF-α), interleukin 1 beta (IL-1β), and interleukin 6 (IL-6), and production of nitric oxide, heat, and pain derived from inducible nitric oxide synthase (*i*NOS) and cyclooxygenase-2 (COX-2) [24]. Since excessive exposure to proinflammatory proteins enhances the degradation of lung epithelium [3,25], it is important to find reagents that can alleviate the stimulated NF-κB pathways of acute lung injury patients.

*Angiopteris cochinchinensis* de Vriese (Marattiaceae family) is an evergreen fern mainly located in Vietnam and China. A. cochinchinensis has huge fronds 1–2.5 m long, showing a distinct central vein connected to 10–15 lateral veins. The leaves contain 10–15 pairs of pinnules with a size of 20–50 cm^2^. Genus *Angiopteris* exhibits numerous therapeutic potentials in that *Angiopteris evecta* has shown antibacterial activity against *Bacillus cereus*, *Staphylococcus albus*, and *Salmonella typhi* [26], and *Angiopteris helferiana* has been reported to have anti-inflammatory and antiadipogenic activities [27]. The therapeutic activities of genus *Angiopteris* are mainly derived from a glucoside named angiopteroside [28]. However, no further clinical studies have examined the possibility of therapeutical uses of *A. cochinchinensis* de Vriese. Therefore, we evaluated the anti-inflammatory effects of an ethanol extract of *A. cochinchinensis* (Ac-EE) in lung infections. The RAW264.7 murine macrophage cell line and peritoneal macrophages were used, along with an LPS-induced acute lung injury mouse model to demonstrate the protective effects of Ac-EE against inflammation in lung tissue.

## 2. Results

### 2.1. Phytochemical Components of Ac-EE

**Figure 1:**
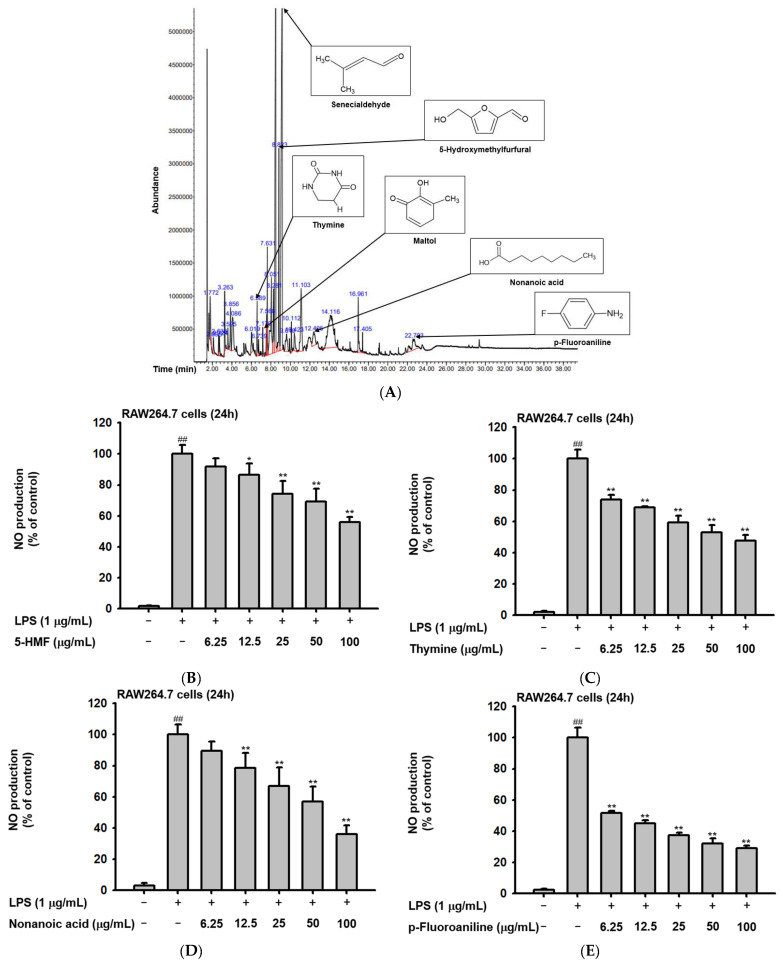
*Angiopteris cochinchinensis* harbors various anti-inflammatory phytochemicals. (**A**) Phytochemical properties of *A. cochinchinensis* were analyzed by GC-MS chromatogram with 28 peaks of the compounds detected. (**B**–**E**) NO scavenging activity of 5-HMF, thymine, nonanoic acid, and *p*-fluoroaniline was evaluated. ##, *p* less than 0.01 relative to the groups without LPS treatment; *, *p* less than 0.05 and **, *p* less than 0.01 relative to the groups with LPS induction.

The phytochemical composition of Ac-EE was analyzed by gas chromatography–mass spectrometry (GC-MS) (Figure 1A). By dividing the corrected percentage peak area by the sum of the corrected areas, we can obtain the total content of each compound in Ac-EE. The most abundant compound was 2-butenal, 3-methyl-, which is also known as senecialdehyde. Table 1 shows all 28 compounds contained in Ac-EE. Interestingly, anti-inflammatory phytochemicals, such as maltol and 5-hydroxymethylfurfural (5-HMF), also are found in Ac-EE [29,30]. In particular, 5-HMF is known to mitigate inflammatory lung injury via attenuation of proinflammatory cytokine production [31]. Surprisingly, NO assay of each of the compounds in Ac-EE showed that other compounds, such as thymine, nonanoic acid, and p-fluoroaniline, also have a NO scavenging effect even better than 5-HMF (Figure 1B–D). This suggests the therapeutic potential of Ac-EE for inflammatory diseases, so we designed further experiments to evaluate the anti-inflammatory response to Ac-EE.

### 2.2. Ac-EE Alleviates Inflammation in Both Macropahge and Acute Lung Injury Mouse Model

**Figure 2:**
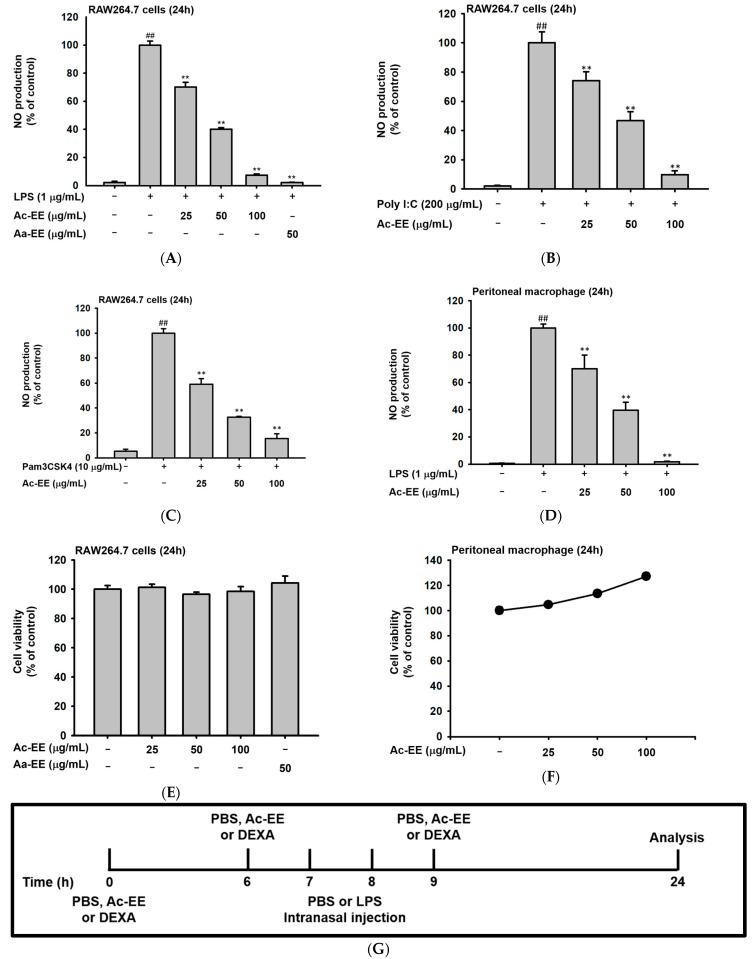

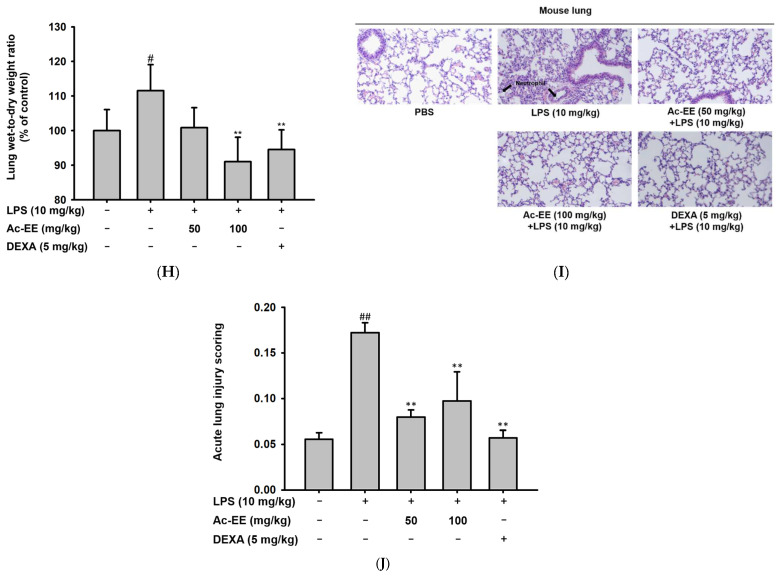
Suppressive effects of Ac-EE on NO production in murine macrophages and lung inflammation in an acute lung injury mouse model. (**A**–**C**) NO synthesis was measured in RAW264.7 cells stimulated by LPS (1 μg/mL), Poly (I:C) (200 μg/mL), or Pam3CSK4 (10 μg/mL) in the presence (25–100 μg/mL) or absence of Ac-EE. (**D**) NO production from LPS-activated peritoneal macrophages was detected with different concentrations of Ac-EE. (**E**,**F**) Cytotoxicity levels of Ac-EE in RAW264.7 cells and peritoneal macrophages were determined. (**G**) Overview of the in vivo acute lung injury mouse model with LPS induction. (**H**) The degree of lung edema was calculated by measuring the wet-to-dry weight ratio. (**I**) Histological photographs of pulmonary tissues observed under a microscope after H&E staining. Black arrows show neutrophils in lung tissues. (**J**) Lung injury score was measured by counting the number of neutrophils, the generation of hyaline membrane, and alveolar septal thickening. #, *p* less than 0.05 and ##, *p* less than 0.01 relative to the normal group; and **, *p* less than 0.01 relative to the LPS induction or vehicle group.

To evaluate the anti-inflammatory effects of Ac-EE, nitric oxide (NO) assays of RAW264.7 cells and peritoneal macrophages were conducted. RAW264.7 cells were activated by LPS, Poly (I:C), and Pam3CSK4. For LPS induction, an ethanol extract of *Artemisia asiatica* (Aa-EE), a previously reported anti-inflammatory agent [32], was used for comparison. Treatment with 100 μg/mL of Ac-EE decreased NO production to 7% compared to the 100% positive control group (Figure 2A). RAW264.7 cells activated by Poly (I:C) and Pam3CSK4 also secreted less NO after Ac-EE treatment (Figure 2B,C). Peritoneal macrophages collected from C57BL/6 mice were also stimulated by LPS to measure the inhibitory effect of Ac-EE on NO. The Ac-EE treatment dose-dependently lowered the NO secretion of peritoneal macrophages (Figure 2D). Since decreased NO production might be derived from the cytotoxicity of Ac-EE on macrophages, a cell viability assay was also conducted. Up to 100 μg/mL of Ac-EE treatment showed no cytotoxicity in either RAW264.7 cells or peritoneal macrophages (Figure 2E,F).

The LPS-induced acute lung injury mouse model was designed to reveal the protective effects of Ac-EE against ALI in lung tissue. Oral administration of 50 or 100 mg/kg of Ac-EE was performed twice before intranasal injection of 10 mg/kg of LPS. An additional Ac-EE injection was conducted 1 h after LPS administration (Figure 2G). The most noticeable pathological feature of ALI is pulmonary edema, measured by calculating ratio between wet and dry weight of the left lung tissue. LPS treatment caused a 1.12-fold increase in the ratio compared to that of the normal group (Figure 2H). Due to Ac-EE treatment, pulmonary edema was decreased successfully. Interestingly, administration of 100 mg/kg of Ac-EE seemed to be more effective for decreasing pulmonary edema compared with dexamethasone, the positive control drug [33].

Histological data from H&E staining from the middle and inferior lobes of the mouse lung were collected to assess the degree of lung injury (Figure 2I). Based on the scoring system of the American Thoracic Society (Table 2), the numbers of neutrophil infiltrations into the alveolar and interstitial space, the numbers of hyaline membranes, and septal thickening of the alveolar wall were counted utilizing a microscope. An approximately threefold increase in lung injury score was shown by LPS induction compared with the normal group, but Ac-EE injection (50 or 100 mg/kg) significantly ameliorated the lung injury (Figure 2J).

### 2.3. Ac-EE Reduces LPS-Induced Expression and Secretion of Proinflammatory Mediators

**Figure 3:**
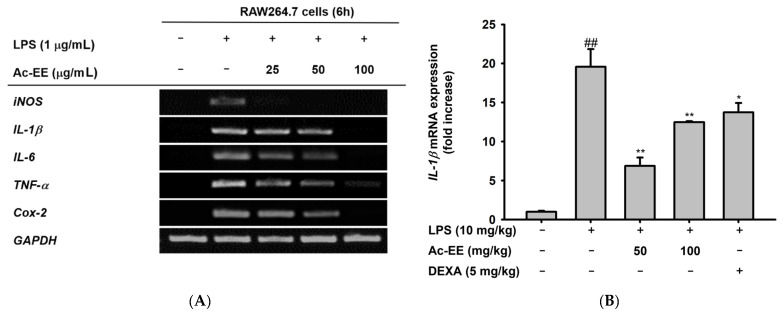

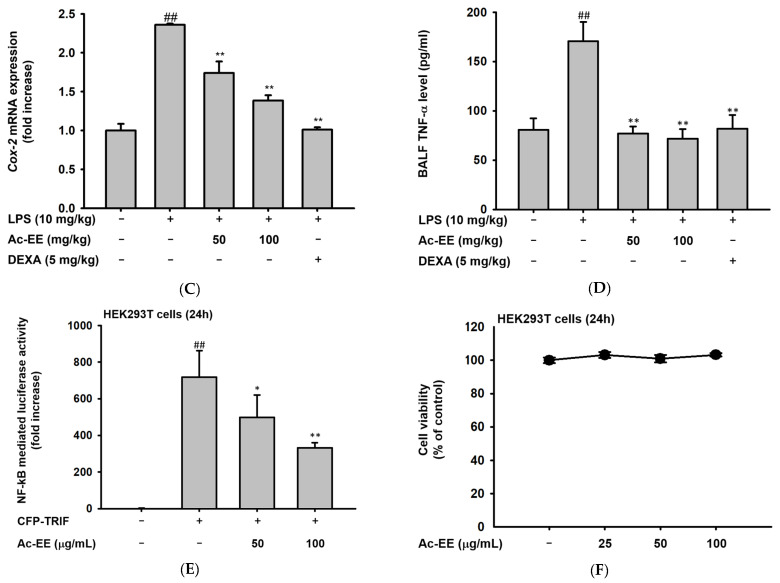
Efficacy of Ac-EE in downregulating the mRNA expression levels and protein levels of proinflammatory cytokines in vitro and in vivo. (**A**) Semiquantitative RT-PCR showing the mRNA expression levels of *i*NOS, COX-2, TNF-α, IL-1β, and IL-6 after 6 h of LPS treatment in Ac-EE-treated RAW264.7 cells. (**B**,**C**) Quantitative real-time PCR indicating the mRNA expression levels of IL-1β and COX-2 in the superior and post-caval lobes of pulmonary tissue. (**D**) Enzyme-linked immunosorbent assay was performed to measure TNF-α in BALF. (**E**) The NF-κB-mediated luciferase reporter activity was measured in HEK293T cells after co-transfection of NF-κB luciferase reporter, CFP-TRIF, and β-galactosidase plasmids. (**F**) Cytotoxicity against HEK293T cells was determined at several concentrations of Ac-EE. ##, *p* less than 0.01 relative to the normal group or the group not treated with CFP-TRIF plasmid; *, *p* less than 0.05 and **, *p* less than 0.01 relative to LPS induction group or the CFP-TRIF-plasmid-treated group.

To elucidate the specific defense mechanism of Ac-EE against ALI, mRNA expression levels of several proinflammatory genes were measured with semi-quantitative RT-PCR and quantitative real-time PCR. RAW264.7 cells were pretreated with 25–100 μg/mL of Ac-EE and activated with 1 μg/mL of LPS. Surprisingly, 100 μg/mL of Ac-EE reduced the mRNA expression levels of iNOS, IL-1β, IL-6, TNF-α, and COX-2 to a degree that the bands were almost invisible (Figure 3A). The mRNA expression levels of proinflammatory factors in the superior and post-caval lobes of the lung were also detected. IL-1β and COX-2 mRNA expression were inhibited by both 50 and 100 mg/kg of Ac-EE treatment (Figure 3B,C). Moreover, TNF-α secretion in bronchoalveolar lavage fluid was analyzed by ELISA to confirm the inhibitory effect of Ac-EE on cytokine secretion. Both concentrations (50 and 100 μg/mL) of Ac-EE downregulated the TNF-*α* secretion to the normal level (Figure 3D). In summary, the expression and secretion of proinflammatory mediators were attenuated by Ac-EE treatment.

For further explanation of the therapeutic potential of Ac-EE, we aimed to identify the signaling pathway involved in the anti-inflammatory effects of Ac-EE. Therefore, a luciferase reporter assay was conducted to determine the specific transcriptional factor regulated by Ac-EE. As NF-κB is one of the most well-known transcription factors that promotes proinflammatory gene expression, we assessed the suppressive effect of Ac-EE on the NF-κB promoter activity level with a luciferase assay. In the experiment, co-transfection of TRIF gene, the NF-κB-Luc gene, and β-galactosidase gene is conducted in HEK293T cells. The NF-κB luciferase activity at 100 μg/mL of Ac-EE decreased by nearly half compared to that observed in the absence of Ac-EE treatment (Figure 3E). In addition, 25 to 100 μg/mL of Ac-EE showed no cytotoxicity in HEK293T cells (Figure 3F).

### 2.4. Ac-EE Inhibits NF-κB Signaling Pathway via Targeting Src

**Figure 4:**
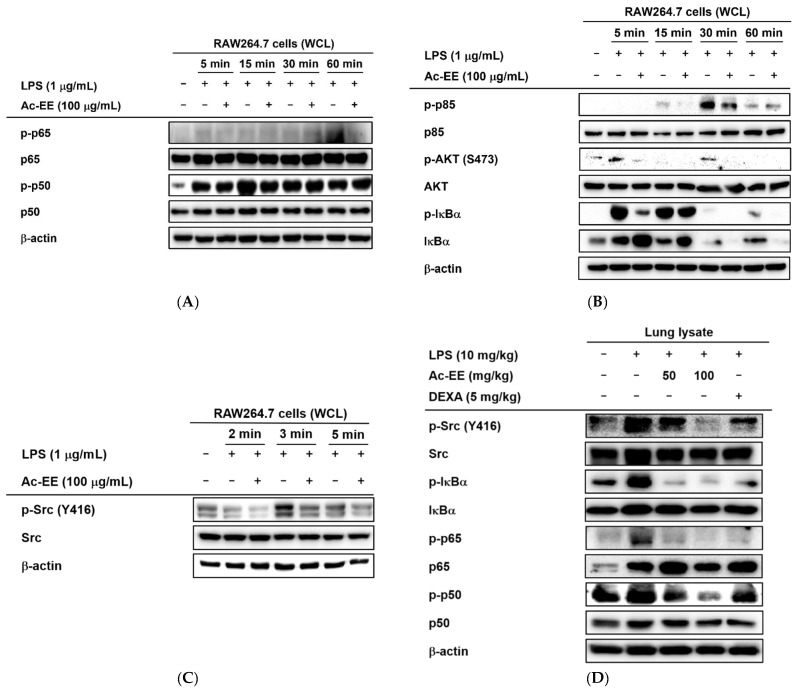

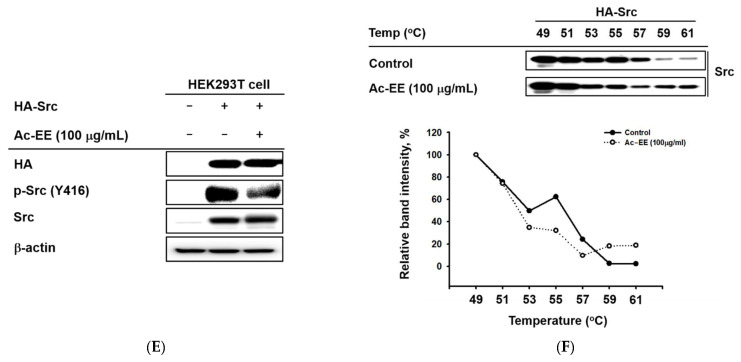
Strategies for finding the target of Ac-EE in anti-inflammatory responses. (**A**) The phosphorylation levels of NF-κB subunits and the protein expression levels of the total forms of the subunits were detected in whole cell lysates of RAW264.7 cells activated by 5 to 60 min of treatment with LPS after 30 min of pretreatment with 100 μg/mL Ac-EE. (**B**) Phosphorylation of the members of the NF-κB signaling pathway, p85, AKT, and IκBα, upon 5 to 60 min of LPS stimulation, was assessed by Western blot analysis. (**C**) The phosphorylation and total protein levels of protein kinase Src were confirmed after 2 to 5 min of LPS induction. (**D**) Western blotting analysis on the superior and post-caval lobes from LPS-treated mice indicates the phosphorylation and total protein expression levels of p50, p65, IκBα, and Src. (**E**) The phosphorylation level of Src in HA-Src-transfected HEK293T cells was detected by Western blot. (**F**) Cellular thermal shift assay was performed to confirm the direct interaction between Src and Ac-EE.

Western blotting analysis showed the suppressive effects of Ac-EE on the phosphorylation of proteins involved in the NF-κB signaling cascade. Phosphorylation of NF-κB family members p50 and p65 allows translocation of the NF-κB complex to the nucleus for further transcriptional activities. However, Ac-EE treatment strongly inhibited the phosphorylation of p65 at 60 min after LPS stimulation. The phosphorylation of p50 was also disrupted, especially at 15 min after LPS treatment (Figure 4A).

For upstream molecules related to the NF-κB pathway, 30 min of pretreatment with Ac-EE clearly decreased the levels of phospho-IκBα, AKT, and p85 within 60 min of LPS exposure (Figure 4B). The phosphorylation of Src, an upstream molecule of the proteins mentioned above, was also reduced by Ac-EE treatment from 2 min to 5 min after LPS induction (Figure 4C). Measurement of the protein levels from the lung tissues of the acute lung injury model showed results consistent with previous experiments in that the phosphorylation of the NF-κB subunits (p50 and p65) and upstream proteins (IκBα and Src) was lowered by oral administration of Ac-EE in a concentration-dependent manner (Figure 4D).

To further determine the main target of Ac-EE, Src was overexpressed in HEK293T cells for cellular thermal shift assays (CETSAs) [34]. Consequently, Ac-EE exhibited inhibitory effects on the phosphorylation of Src in HEK293T cells (Figure 4E). Interestingly, Ac-EE imparted thermal stability to Src, especially at relatively high temperatures (59 °C and 61 °C) (Figure 4F). These results indicate that Ac-EE attenuates acute lung injury by directly targeting Src to inhibit subsequent inflammatory responses.

## 3. Discussion

ALI is a growing concern in humans and often leads to acute respiratory distress syndrome (ARDS) with high morbidity and mortality [35]. It was reported that ALI led to the death of about 40% of ALI patients in the United States in 2005 [36]. Since the impact of ALI emphasizes the importance of treatment options for this disease, various therapeutic agents (corticosteroids, nonsteroidal anti-inflammatory drugs, antioxidants, etc.) have been tested [37]. However, the complexity of the pathological process and the diversity of therapeutic surroundings have limited studies to the late or terminal events of ALI, neglecting the early pathological features, such as interstitial edema and devastation of alveolar cells [38,39]. Therefore, a new treatment that can directly target specific stages of ALI occurrence should be introduced, and the specific therapeutic mechanism of the drug should be revealed.

The genus *Angiopteris*, which is in the family Marattiaceae, has pinnately divided leaves with developed fleshy stipules. Various species of *Angiopteris*, including *Angiopteris evecta*, which has antibacterial properties [40], have shown therapeutic activity. However, no further study has been conducted to determine a clinical approach to treatment with *Angiopteris cochinchinensis*. Previous GC-MS analysis showed that *A. cochinchinensis* harbors phytochemicals, including maltol and 5-hydroxymethylfurfural, which act as strong anti-inflammatory agents [29,41]. The extract was also composed of molecules including nonanoic acid, p-fluoroaniline, and thymine, which reduce NO production in LPS-treated RAW264.7 cells. Therefore, we conducted experiments to assess whether an ethanol extract of *A. cochinchinensis* mitigates ALI.

Fungal, viral, and bacterial infections are the main causes of ALI [42]. Specifically, Gram-negative bacteria, such as *P. aeruginosa*, secrete various endotoxins, including LPS [43]. Exposure to LPS accelerates neutrophil recruitment and expression of proinflammatory molecules [44]. The outcomes derive from the interaction between LPS and TLR4, consequently activating inflammatory responses, such as the NF-κB signaling pathway [45]. Our study successfully established in vitro experiments with murine macrophages and an ALI mouse model induced by LPS to evaluate the protective effects of Ac-EE against ALI.

In the screening step, Ac-EE decreased NO synthesis in mouse-derived macrophages upregulated by LPS treatment. The inhibitory effects of Ac-EE upon NO synthesis were confirmed in Poly (I:C)- and Pam3CSK4-treated RAW264.7 cells. Since NO acts as a signaling molecule that induces inflammation after its excessive production derived from pathogen infection, we expected Ac-EE to have an anti-inflammatory effect. Therefore, the alleviating effects of Ac-EE on ALI were additionally assessed with animal experiments. Ac-EE not only attenuated pulmonary edema, but also alleviated the lung injury shown in histological data. To be specific, the number of neutrophils in both the alveolar space and the interstitial space declined after Ac-EE administration, and Ac-EE prevented the formation of hyaline membranes and the septal thickening of alveolar cells, the main histological hallmarks in the exudative phase of ARDS [46]. These results demonstrate that Ac-EE can ameliorate acute lung injury through anti-inflammatory responses.

Next, the suppressive effects of Ac-EE on the mRNA expression of *i*NOS, which produces NO; COX-2, which produces proinflammatory factor PGE2; and proinflammatory cytokines, such as TNF-α, IL-6, and IL-1β, were evaluated by semiquantitative RT-PCR analysis in RAW264.7 cells. After Ac-EE treatment, the mRNA levels of COX-2 and IL-1β in the lung tissue decreased. The cytokine levels in bronchoalveolar lavage fluid (BALF) are usually measured to demonstrate the degree of lung inflammation, so TNF-α concentration was additionally measured there using ELISA [47]. TNF-α secretion in BALF decreased to normal level after Ac-EE treatment. These results indicate that the protective effect of Ac-EE on lung tissue against inflammation is derived from downregulation of expression and secretion of proinflammatory mediators.

In the luciferase reporter assay, the activity of NF-κB, one of the most representative transcription factors participating in the inflammatory response, was reduced by Ac-EE. Moreover, LPS-induced NF-κB subunit activation was decreased by Ac-EE treatment. Thus, we assessed the phosphorylation of IκBα, AKT, p85, and Src, which are the upstream molecules of the NF-κB signaling pathway, in RAW264.7 cells. The phosphorylation of IκBα, AKT, p85, and Src was strongly reduced by Ac-EE. In the acute lung injury mouse model, phosphorylation of the proteins involved in the NF-κB signaling pathway, including the subunits of NF-κB p50 and p65, were decreased after administration of Ac-EE. These results indicate Ac-EE as a candidate drug with a direct target. Western blotting analysis using an overexpression strategy showed the inhibitory effect of Ac-EE on the phosphorylation of transfected Src in HEK293T cells. Next, CETSA analysis was conducted to assess the direct interaction of Src and Ac-EE in HEK293T cells.

Conventional anti-inflammatory drugs include corticosteroid agents, such as dexamethasone [48]. Although it shows intense mitigation of lung injury, as in results, dexamethasone is known to induce muscle atrophy with activation of mechanistic target of rapamycin (mTOR) pathway and Connexin 43 hemichannels (Cx HCs) [49]. Nonsteroid anti-inflammatory drugs (NSAIDs) also exert cytotoxicity by inducing oxidative stress in mitochondria, which accompanies serious organ damage [50]. Moreover, NSAIDs have weak applicational range in curing inflammatory diseases, since most NSAIDs mainly narrow down their targets to cyclooxygenase family (COX) [51]. Therefore, botanical medicines harboring bioactive phytochemicals are emerging as possible alternatives. Here, we found novel interaction of Src, an upstream molecule of NF-κB pathway, and Ac-EE, which results in significant alleviation of ALI to a normal level. Thus, we expect that Ac-EE can be an effective therapeutic agent that can control progression of serious respiratory inflammation.

## 4. Materials and Methods

### 4.1. Materials and Reagents

RAW264.7 cells (ATCC TIB-71) and HEK293T cells (ATCC CRL-1573) were purchased from the American Type Culture Collection (Rockville, MD, USA). Penicillin, streptomycin, fetal bovine serum (FBS), Roswell Park Memorial Institute 1640 (RPMI 1640) medium, Dulbecco’s modified Eagle’s medium (DMEM), and Opti-MEM™ Reduced Serum Medium were obtained from GIBCO (Grand Island, NY, USA). Lipopolysaccharide (LPS), Poly (I:C), Pam3CSK4, MTT, polyethylenimine (PEI), dimethyl sulfoxide (DMSO), and dexamethasone were bought from Sigma Chemical Co. (St. Louis, MO, USA). Phosphate-buffered saline (PBS) was obtained from Samchun Pure Chemical Co. (Gyeonggi-do, Republic of Korea). TRI Reagent^®^ solution was purchased from Molecular Research Center, Inc. (Cincinnati, OH, USA). The cDNA synthesis kit was obtained from Thermo Fisher Scientific (Waltham, MA, USA). Primers for semiquantitative RT-PCR and quantitative rea-time PCR were synthesized from Macrogen (Seoul, Korea), and PCR premix was purchased from Bio-D Inc. (Seoul, Korea). The ELISA kit for measuring TNF-α protein level was from R&D Systems (Minneapolis, MN, USA). Total and phospho-p50, p65, IκBα, AKT, p85, and Src targeting antibodies were purchased from Cell Signaling Technology (Beverly, MA, USA). Antibody against β-actin was purchased from Santa Cruz Biotechnology, Inc. (Santa Cruz, CA, USA).

### 4.2. Ac-EE Preparation and Gas Chromatography–Mass Spectrometry

An ethanol extract of the leaves of *Angiopteris cochinchinensis* de Vriese (Ac-EE) was received from the National Institute for Biological Resources (Incheon, Korea) belonging to the Ministry of Environment. The leaves of *A. cochinchinensis* were ground and dried before infiltration of 70% EtOH for 1 day at RT. The extract was then filtered in vacuum state at 40 °C under 10 hPa. After further enrichment with a rotary flash evaporator (N-1000SWD, EYELA), the extract was lyophilized. Prepared extract was resolved in DMSO to make 100 mg/mL stock solution for in vitro experiments. The stock was diluted with each cell culture medium for treatment. In the animal experiments, Ac-EE was dissolved in PBS to prepare the target concentration (100 mg/kg). Gas chromatography–mass spectrometry (GC-MS) analysis was introduced with the help of the Cooperative Center for Research Facilities in SKKU (Suwon, Republic of Korea).

### 4.3. Cell Culture

Mouse-derived RAW264.7 macrophage cell line and human-derived HEK293T embryonic kidney cell line were used in the experiments. The cells were, respectively, cultured in RPMI 1640 medium and DMEM medium under the condition of 37 °C and 5% CO_2_. Both of the media contained additional 10% FBS and 1% penicillin/streptomycin. The cells were sub-cultured once every two to three days, when the confluency reached 80–90%.

### 4.4. Isolation of Peritoneal Macrophages

First, 1 mL of sterile 4% thioglycolate broth was administered to male C57BL/6 mice via intraperitoneal (IP) injection. Peritoneal macrophages were collected through IP lavage four days after the injection. After an additional washing process with Blood Cell Lysis Buffer (Sigma), the isolated peritoneal macrophages were seeded in a 96-well plate with a concentration of 1 × 10^6^ cells/mL in 10% FBS and 1% antibiotics complemented RPMI 1640 at 37 °C in 5% CO_2_.

### 4.5. In Vivo LPS-Induced Acute Lung Injury Mouse Model

Five-week-old male C57BL/6 mice were classified into 5 groups (normal, vehicle, Ac-EE 50 mg/kg, Ac-EE 100 mg/kg, and dexamethasone; *n* = 5). Animals were acclimatized at 22–24 °C and a 12 h light/dark cycle with free access to food and water. The experiment was based on the guidelines from the Institutional Animal Care and Use Committee at Sungkyunkwan University (SKKUIACUC2021-09-54-1). Vehicle (DMSO), 50 and 100 mg/kg of Ac-EE, and 5 mg/kg of dexamethasone were orally administered 7 and 1 h before the intranasal administration of 10 mg/kg of LPS (dissolved in PBS 50 μL). One hour after the LPS injection, the last oral administration of Ac-EE or dexamethasone was conducted. After 16 h of induction, all mice were anesthetized using isoflurane and sacrificed. BALF was collected immediately, and every lobe of each lung was obtained. BALF was used for ELISA, while the left lobe was used to analyze the wet-to-dry ratio. The middle and inferior lobes were used for histopathological data, and the superior and post-caval lobes were used for Western blotting and quantitative real-time PCR.

### 4.6. Nitric Oxide Production Assay

RAW264.7 cells or peritoneal macrophages were cultured in 96-well plates at concentrations of 1 × 10^6^ cells/mL. Then, 30 min of pretreatment with 50 μL of Ac-EE or with Aa-EE, a positive control medicine, was conducted. Next, 1 μg/mL of LPS, which activates TLR4, 200 μg/mL of Poly (I:C), which activates TLR3, or 10 μg/mL of Pam3CSK4, which activates TLR1/2, was administered to activate the cells. After 24 h of incubation, 100 μL of the supernatant was collected. The reaction was induced by adding Griess agent 100 μL to the supernatant. Absorbance at 540 nm was measured.

### 4.7. Cell Viability Assay

For the cell viability assay, 1 × 10^6^ cells/mL of RAW264.7 cells, peritoneal macrophages, and HEK293T cells were cultured in 96-well plates and incubated overnight for enough confluency. Then, cells were exposed to Ac-EE (0–100 μg/mL) for 1 day. After treatment, 10 μL of MTT solution were added to the cells, and the cells were cultured for another 3 h. Then, 100 μL of 0.01 M HCl dissolved in 10% SDS was added to stop the reaction. The absorbance of the mixture was evaluated at 540 nm.

### 4.8. Lung Wet-to-Dry Weight Ratio Measurement

The left lobes of lung tissues from 5 mice per group were washed with PBS and drained with Kleenex (Yuhan Kimberly, Sonpa, Seoul, Korea). After recording the wet weight, lung tissues were dried at 80 °C for 72 h to obtain the dry weight, as previously reported [52]. The wet-to-dry ratio was calculated to assess the degree of edema in the lung tissue.

### 4.9. Histological Analysis of Lung Tissue

Middle and inferior parts of the pulmonary lobes from 3 mice per group were collected and fixed in 4% formalin for 48 h. The fixed tissues were all embedded in paraffin and sectioned at 4 μm thick. Hematoxylin and eosin were used to stain the tissues. The lung injury was evaluated by measuring the intensity of septal thickening of alveolar walls, neutrophil infiltration, and formation of membrane structure composed of cell debris.

### 4.10. mRNA Expression Level Measurement Using Semiquantitative RT-PCR and Quantitative Real-Time PCR

RAW264.7 cells pretreated with Ac-EE for 30 min were stimulated by 1 μg/mL of LPS for 6 h. In the in vivo acute lung injury model, parts of the superior and post-caval pulmonary lobes were ground with liquid nitrogen. Total RNA from the cells and the lung tissues were extracted with 300 and 600 μL of TRIzol reagent, respectively. Then, 1 μg of RNA from each sample was used for cDNA synthesis. In semiquantitative RT-PCR, the mRNA expression levels of *i*NOS, COX-2, TNF-α, IL-1β, IL-6, and GAPDH in RAW264.7 cells were measured. In quantitative real-time PCR, the mRNA expression levels of COX-2 and IL-1β were measured relative to that of GAPDH. Primers are listed in Table 3 and Table 4.

### 4.11. ELISA in Bronchoalveolar Lavage Fluid

For BALF analysis, 500 μL of BALF from the in vivo acute lung injury model was collected by inserting a catheter into the trachea of an anesthetized mouse and added to 1 mL of PBS in 100 μm EDTA [53]. Then, the protein level of TNF-α released in BALF was assessed according to the manufacturer’s instructions.

### 4.12. Luciferase Reporter Assay

HEK293T cells were cultured in 24-well plates with a density of 1 × 10^6^ cells/mL and co-transfected with TRIF, which acts as a luciferase gene activator with NF-κB-Luc and the β-galactosidase gene. The transfection was supported by PEI, as described previously [54]. Twenty-four hours later, Ac-EE (0–100 μg/mL) was administered, and cells were incubated for another 24 h. Then, cell lysis was performed by the freeze and thaw method, and the luciferase reporter activity induced by luciferin was measured by detecting luminescence. The luciferase reporter activity was normalized by comparing it with β-galactosidase activity.

### 4.13. Whole Cell Lysate Preparation and Western Blotting Analysis

RAW264.7 cells (1 × 10^6^ cells/mL) and HEK293T cells (3 × 10^5^ cells/mL) were cultured in 6-well plates. In the RAW264.7 cells, Ac-EE and LPS were administered at different time points [55]. HEK293T cells treated with HA-Src plasmid for 24 h are exposed to 100 μg/mL of Ac-EE for another 24 h. The cells were then washed in 1 mL of cold PBS and collected with cell lysis buffer (20 mM Tris-HCl, pH 7.5; 20 mM NaF, 25 mM; β-glycerol phosphate, pH 7.5; 150 mM NaCl; and 2% NP-40 with protease inhibitors (100 mM PMSF, 2 μg/mL leupeptin, pepstatin, 2 μg/mL aprotinin, and 2 mM EDTA)). The lung tissues were lysed by a sonicator (Thermo Fisher Scientific, Waltham, MA, USA) and cell lysis buffer to obtain whole cell lysates. The cell lysates from both in vitro and in vivo experiments were centrifuged at 11,000× *g* for 5 min at 4 °C, and the subsequent supernatant was used for Western blotting analysis.

The protein samples were separated according to size by SDS-polyacrylamide gel electrophoresis and transferred to a polyvinylidene difluoride (PVDF) membrane. Proteins in the PVDF membrane were interacted with specific target antibodies, and immunoreactivity was measured by detecting the band intensity of each protein.

### 4.14. Cellular Thermal Shift Assay

HEK293T cells were seeded onto 6-well plates at a density of 3 × 10^5^ cells/mL. The cells were transfected with HA-Src plasmids for 24 h and classified into two groups: 100 μg/mL Ac-EE or vehicle (DMSO). After an additional 24 h of treatment with Ac-EE or DMSO, the cells were collected with cold PBS and equally aliquoted into seven PCR tubes. The cells were then heated for 3 min at a set temperature (49 to 61 °C) and incubated at RT for 3 min. Cell lysis was performed by three repetitions of a freezing and thawing cycle using LN_2_ [56]. Proteins from the cell lysates were obtained by centrifugation at 12,000 rpm for 30 min and analyzed by Western blotting analysis.

### 4.15. Statistical Analysis

All experiments were conducted with at least three independent samples, and the data are presented as mean ± standard error of mean. All data from in vitro experiments were checked by one-way ANOVA with Tukey’s multiple comparisons test. Statistical significances from in vivo experiments, including lung wet weight/dry weight ratio analysis, acute lung injury score analysis, real-time PCR, and ELISA, were determined by one-way ANOVA with Dunnett’s multiple comparisons test evaluating significant differences with the LPS induction group. GraphPad Prism 8.01 software from GraphPad Software (La Jolla, CA, USA) was utilized to perform calculation. A *p*-value under 0.05 showed statistical significance.

## 5. Conclusions

**Figure 5:**
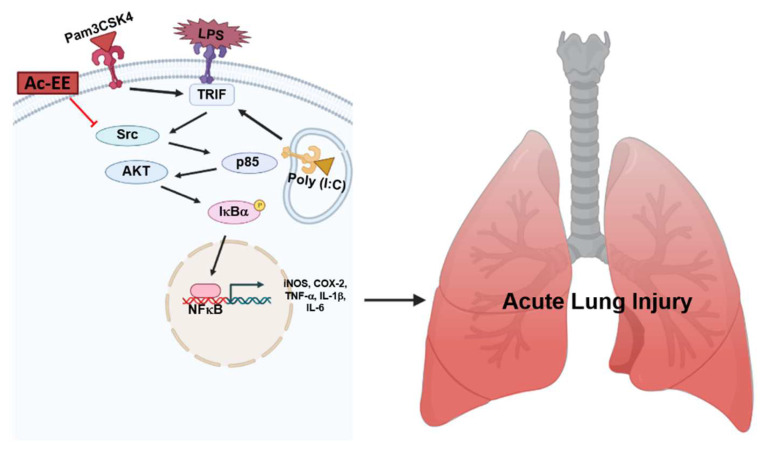
The potential inhibitory pathway of Ac-EE-mediated anti-lung injury activities.

LPS-induced acute lung injury upregulates the recruitment of neutrophils in pulmonary tissue and damages alveolar cells, causing alveolar septal thickening and the formation of hyaline membranes. Pulmonary edema can be also seen in the lung tissue after LPS infection, which can impair gas exchange, eventually leading to severe respiratory failure. However, we revealed that Ac-EE can ameliorate acute lung injury by suppressing proinflammatory mediators, as summarized in Figure 5. Moreover, we demonstrated in both in vitro and in vivo experiments that these effects were derived from the inhibitory role of Ac-EE on the NF-κB signaling pathway. We also revealed that the prime target of Ac-EE is the protein kinase Src, which modulates phosphorylation of downstream molecules, including p50 and p65, which are NF-κB subunits. Therefore, Ac-EE can be considered as a possible herbal medicine attenuating acute lung injury.

## Figures and Tables

**Table 1:** Phytochemical analysis of ethanol extract of Ac-EE.

Peak No.	RT	Name of the Compound	Corrected Area	% of Total
1	1.772	Acetic acid	29216024	1.64
2	2.098	2-Propanone, 1-hydoxy-	12843838	0.72
3	2.638	2-Propenoyl chloride	8113271	0.46
4	2.724	1,2,3-Propanetriol, 1-acetate	11290321	0.63
5	3.263	Glyceraldehyde	34415997	1.93
6	3.595	2-Furanmethanol	10248331	0.57
7	3.856	Dihydro-2(3H)-thiophenone	28723699	1.61
8	4.086	Dihydroxyacetone	52160462	2.93
9	6.019	1,4-Cyclohex-2-enedione	18462433	1.04
10	6.589	Thymine	25694457	1.44
11	6.739	3-Furanacarboxylic acid, methyl ester	8426843	0.47
12	7.170	Maltol	11986818	0.67
13	7.568	Isothiazole, 3-methyl-	26854159	1.51
14	7.631	4H-Pyran-4one, 2,3-dihydro-3,5-dihydroxy-6-methyl-	44906782	2.52
15	8.051	Cyclopentanone ethylene ketal	92735782	5.20
16	8.281	Pyrrolidin-1-acetic acid	44123254	2.47
17	8.492	Divinyl sulfide	330210094	18.52
18	8.823	5-Hydroxymethylfurfural	85088443	4.77
19	9.156	2-Butenal, 3-methyl-	435954466	24.45
20	9.619	4H-Pyran-4-one, 2,3-dihydro-3,5-dihydroxy-6-methyl-	32698466	1.83
21	10.112	Isosorbide	13278795	0.74
22	10.423	Heptane, 2,3-epoxy-	19897179	1.12
23	11.103	Formic acid, hex-2-yl ester	86990006	4.88
24	12.406	Nonanoic acid	33776334	1.89
25	14.116	*N*-Methoxymethyl-*N*-methylacetamide	190707640	10.70
26	16.961	2-Amino-actadecane-1,3,4 triol 1,3:2,4-bis-methaneboronate	39099092	2.19
27	17.405	*n*-Hexadecanoic acid	7408037	0.42
28	22.703	*p*-Fluoroaniline	47763799	2.68

**Table 2:** Scoring lung injury with methods from American Thoracic Society documents.

Measurement Criteria	Score		
	0	1	2
A. Neutrophil infiltration to the interstitial space	Not found	1 to 5	More than 5
B. Neutrophils infiltration to the alveolar space	Not found	1 to 5	More than 5
C. Numbers of hyaline membrane	Not found	3	More than 3
D. Septal thickening of alveolar wall	More than 2×	2 to 4×	More than 4×
Score = [(20 × A) + (14 × B) + (7 × C) + (2 × D)]/(field number × 100)			

**Table 3:** Sequences of primers used for semiquantitative RT-PCR analysis.

Gene Name	Sequence (5′–3′)	
*i*NOS	Forward	TGCCAGGGTCACAACTTTACA
	Reverse	ACCCCAAGCAAGACTTGGAC
COX-2	Forward	TGAGTACCGCAACGCTTCT
	Reverse	TGGGAGGCACTTGCATTGAT
TNF-α	Forward	TTGACCTCAGCGCTGAGTTG
	Reverse	CCTGTAGCCCACGTCGTAGC
IL-1β	Forward	CAGGATGAGGACATGAGCACC
	Reverse	CTCTGCAGACTCAAACTCCAC
IL-6	Forward	GGAAATCGTGGAAATGAG
	Reverse	GCTTAGGCATAACGCACT
GAPDH	Forward	GAAGGTCGGTGTGAACGGAT
	Reverse	AGTGATGGCATGGACTGTGG

**Table 4:** Sequences of primers used for quantitative real-time PCR analysis.

Gene Name	Sequence (5′–3′)	
COX-2	Forward	TTGGAGGCGAAGTGGGTTTT
	Reverse	TGGCTGTTTTGGTAGGCTGT
IL-1β	Forward	GTGAAATGCCACCTTTTACAGTG
	Reverse	CCTGCCTGAAGCTCTTGTTG
GAPDH	Forward	GGAGAGTGTTTCCTCGTCCC
	Reverse	ATGAAGGGGTCGTTGATGGC

## Data Availability

The data are contained within the article.

## Abbreviations

ALI	Acute lung injury
Ac-EE	Ethanol extract of Angiopteris cochinchinensis
PRR	Pattern recognition receptors
PAMP	Pattern-associated molecular patterns
LPS	Lipopolysaccharide
TLR	Toll-like receptors
NF-κB	Nuclear factor kappa B
*i*NOS	Induced nitric oxide synthase
COX-2	Cyclooxygenase-2
IL-1β	Interleukin-1 beta
IL-6	Interleukin-6
TNF-α	Tumor necrosis factor alpha
PI3K	Phosphoinositide 3-kinase
GC-MS	Gas chromatography–mass spectrometry
CETSA	Cellular thermal shift assay
